# Urinary bladder cancer as a late sequela of traumatic spinal cord injury

**DOI:** 10.1186/s40779-021-00322-7

**Published:** 2021-04-29

**Authors:** Ralf Böthig, Christian Tiburtius, Wolfgang Schöps, Michael Zellner, Oliver Balzer, Birgitt Kowald, Sven Hirschfeld, Roland Thietje, Aki Pietsch, Ines Kurze, Martin Forchert, Thura Kadhum, Klaus Golka

**Affiliations:** 1 Department of Neuro-Urology, Centre for Spinal Cord Injuries, BG Klinikum Hamburg, 21033 Hamburg, Germany; 2 Urological Practice, 53757 Sankt Augustin, Germany; 3 Department of Urology and Neuro-Urology, Johannesbad Fachklinik, 94072 Bad Füssing, Germany; 4 Biomechanical Laboratory, Centre for Spinal Cord Injuries, BG Klinikum Hamburg, 21033 Hamburg, Germany; 5 Centre for Spinal Cord Injuries, BG Klinikum Hamburg, 21033 Hamburg, Germany; 6 Department of Sports and Rehabilitation Medicine, BG Klinikum Hamburg, 21033 Hamburg, Germany; 7 Department of Paraplegiology and Neuro-Urology, Centre for Spinal Cord Injuries, Zentralklinik Bad Berka, 99437 Bad Berka, Germany; 8 Staff Position Accident Insurance Law, Statutory Accident Insurance for Wood and Metal (BGHM), 33602 Bielefeld, Germany; 9 Department of Psychosomatic Rehabilitation, Mittelrheinklinik Fachklinik, 56154 Boppard-Bad Salzig, Germany; 10 Clinical Occupational Medicine, Leibniz Research Centre for Working Environment and Human Factors at TU Dortmund (IfADo), 44139 Dortmund, Germany

**Keywords:** Traumatic spinal cord injury, Neurogenic bladder, Transitional cell carcinoma, Squamous cell carcinoma, Survival time, Battlefield injury, Medical assessment

## Abstract

**Background:**

Traumatic spinal cord injury (SCI) is also a combat-related injury that is increasing in modern warfare. The aim of this work is to inform medical experts regarding the different course of bladder cancer in able-bodied patients compared with SCI patients based on the latest medical scientific knowledge, and to present decision-making aids for the assessment of bladder cancer as a late sequela of traumatic SCI.

**Methods:**

A study conducted between January 1998 and December 2019 in the BG Trauma Hospital Hamburg formed the basis for the decision-making aids. Urinary bladder cancer was diagnosed in 40 out of 7396 treated outpatient and inpatient SCI patients. General patient information, latency period, age at initial diagnosis, type of bladder management and survival of SCI patients with bladder cancer were collected and analysed. T category, grading and tumour entity in these patients were compared with those in the general population. Relevant bladder cancer risk factors in SCI patients were analysed. Furthermore, relevant published literature was taken into consideration.

**Results:**

Initial diagnosis of urinary bladder cancer in SCI patients occurs at a mean age of 56.4 years (SD ± 10.7 years), i.e.*,* approximately 20 years earlier as compared with the general population. These bladder cancers are significantly more frequently muscle invasive (i.e., T category ≥ T2) and present a higher grade at initial diagnosis. Furthermore, SCI patients show a significantly higher proportion of the more aggressive squamous cell carcinoma than that of the general population in areas not endemic for the tropical disease schistosomiasis. Consequently, the survival time is extremely unfavourable. A very important finding, for practical reasons is that, in the Hamburg study as well as in the literature, urinary bladder cancer is more frequently observed after 10 years or more of SCI. Based on these findings, a matrix was compiled where the various influencing factors, either for or against the recognition of an association between SCI and urinary bladder cancer, were weighted according to their relevance.

**Conclusions:**

The results showed that urinary bladder cancer in SCI patients differs considerably from that in able-bodied patients. The presented algorithm is an important aid in everyday clinical practice for assessing the correlation between SCI and bladder cancer.

**Supplementary Information:**

The online version contains supplementary material available at 10.1186/s40779-021-00322-7.

## Background

Traumatic spinal cord injury (SCI) is a severe combat-related injury that is increasing in modern warfare. In contrast to previous wars, the percentage of SCIs due to explosive devices, compared to bullet related injuries, has increased [1]. A publication based on figures from the wars in Afghanistan and Iraq reported that between 2003 and 2011, 4% of 1547 orthopaedic injuries presented as SCI. Consequently, an expert consensus on the treatment of combat-related SCIs on the battlefield [1], as well as guidelines for veterans with SCIs (USA), have been released [2].

The initial diagnosis of bladder cancer in patients with SCI is, on average, 20 years earlier than in able-bodied patients in the general population. Furthermore, at initial diagnosis, these tumours are commonly already more aggressive and more advanced. These findings, which have been described in the international literature [3, 4], have been until today, due to a lack of recommendations for medical experts in guidelines, not applied in medical assessments of patients with SCI.

The aim of this work is to present the current scientific evidence in the field of SCI and subsequent bladder cancer, to derive a suggestion for a recommendation for the assessment of a possible causal relationship between bladder cancer and SCI in military medicine, and in nonmilitary related injuries, and to draw the attention of the treating physicians to the huge differences in the course of bladder cancer disease between able-bodied patients and SCI-patients. The present article is based on data published in the literature [3, 4] and the findings of a large ongoing study conducted in the BG Klinikum Hamburg [5–7], including suggestions for the assessment of a causal relationship between bladder cancer and SCI in occupational settings and the suggestion of a period of time after initial SCI diagnosis after that bladder cancer prevalence increases.

## Methods

During the period from 1 January 1998 to 31 December 2019, 7396 SCI patients (2059 females and 5337 males) were treated as outpatients or inpatients in the Centre for Spinal Cord Injuries of the BG Klinikum Hamburg, Hamburg, Germany. This hospital is run by the German Social Accident Insurance and provides more than 120 beds for patients with SCI primarily as a result of occupational and non-occupational related accidents.

General patient information, latency period, age at initial diagnosis, type of bladder management and survival of SCI patients with bladder cancer were collected and analysed. T category, grading and tumour entity (transitional cell carcinoma and squamous cell carcinoma) in these patients were compared with those in the general population. Relevant bladder cancer risk factors in SCI patients were analysed and compared with data from the literature.

The urinary bladder cancer data for the German population were obtained from the German Centre for Cancer Registry Data at Robert Koch Institute (RKI) in Berlin, Germany.

All applicable institutional and governmental regulations concerning the ethical use of the data were observed. The approving institutional review board was the Institution for Statutory Accident Insurance and Prevention in the Health and Welfare Services (address: Pappelallee 33, 22089 Hamburg, Germany, Date 22 June 2015) and the Ethics Committee of the University of Lübeck (AZ 17-345A), Lübeck, Germany.

## Results

**Fig. 1:**
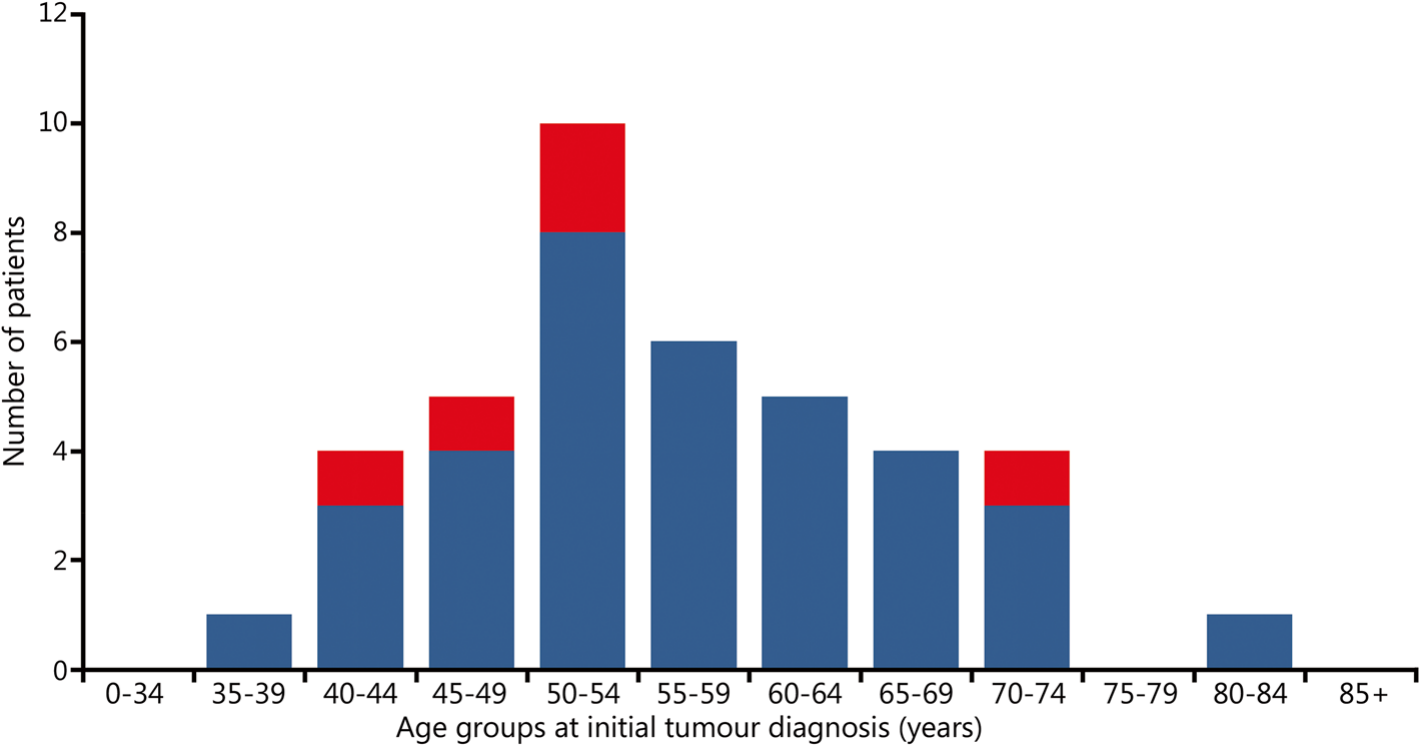
Age and gender of 40 spinal cord injury patients at initial bladder cancer diagnosis. Blue. Male; Red. Female

**Fig. 2:**
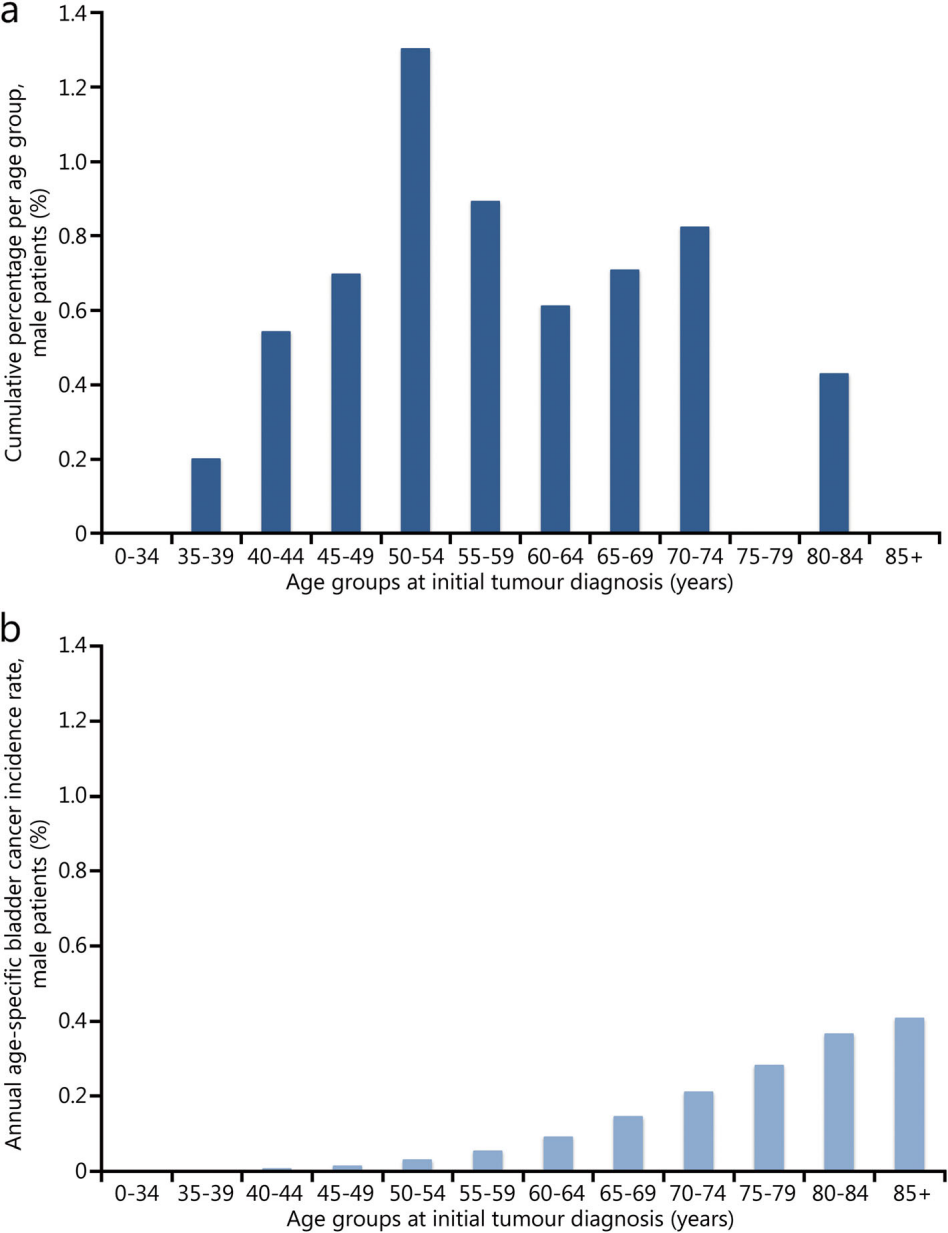
Comparison of cumulative percentage of bladder cancer in spinal cord injury patients and bladder cancer incidence rates in the general population in Germany. **a** Cumulative percentage of bladder cancer in spinal cord injury patients (Hamburg data 1998–2019). **b** Bladder cancer incidence rates in the general population in Germany (Robert Koch Institute data 1999–2016)

**Fig. 3:**
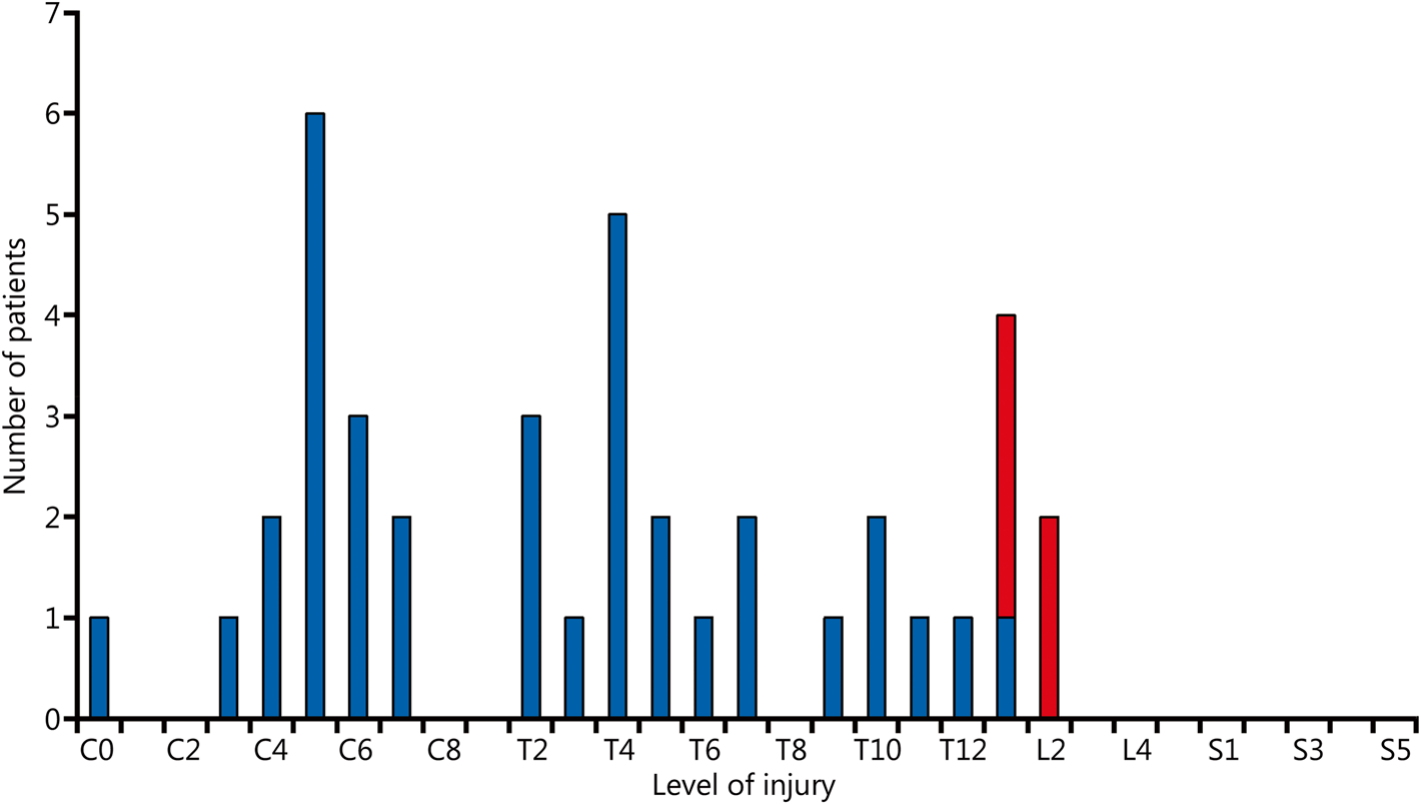
Level of spinal cord injury. Blue. Upper motor neuron lesions (UMNL); Red. Lower motor neuron lesions (LMNL). Please note that the split blue and red bar for L1 indicates one UMNL case and three LMNL cases

The study population consisted of 40 SCI patients (35 males and 5 females) with bladder cancer. The median follow-up of these SCI patients with bladder cancer was 89.5 months. The median age at the time of diagnosis of bladder cancer was 54.5 years for the whole group, 55.0 years for males and 51.0 years for females (Figs. 1 and 2). Thirty-five of 40 patients suffered from a spinal cord lesion at L1 or above with urodynamically proven detrusor overactivity (upper motor neuron lesion, UMNL), while 5 patients (all male) had a spinal cord lesion below L1 with acontractile detrusor function (lower motor neuron lesion, LMNL) (Fig. 3). Twenty SCI cases were due to accidents that were covered by the Statutory Accident Insurance of Germany, including workplace or commuting accidents; 17 were due to accidents not covered by Statutory Accident Insurance of Germany including private accidents; and 3 were due to other injuries.

**Fig. 4:**
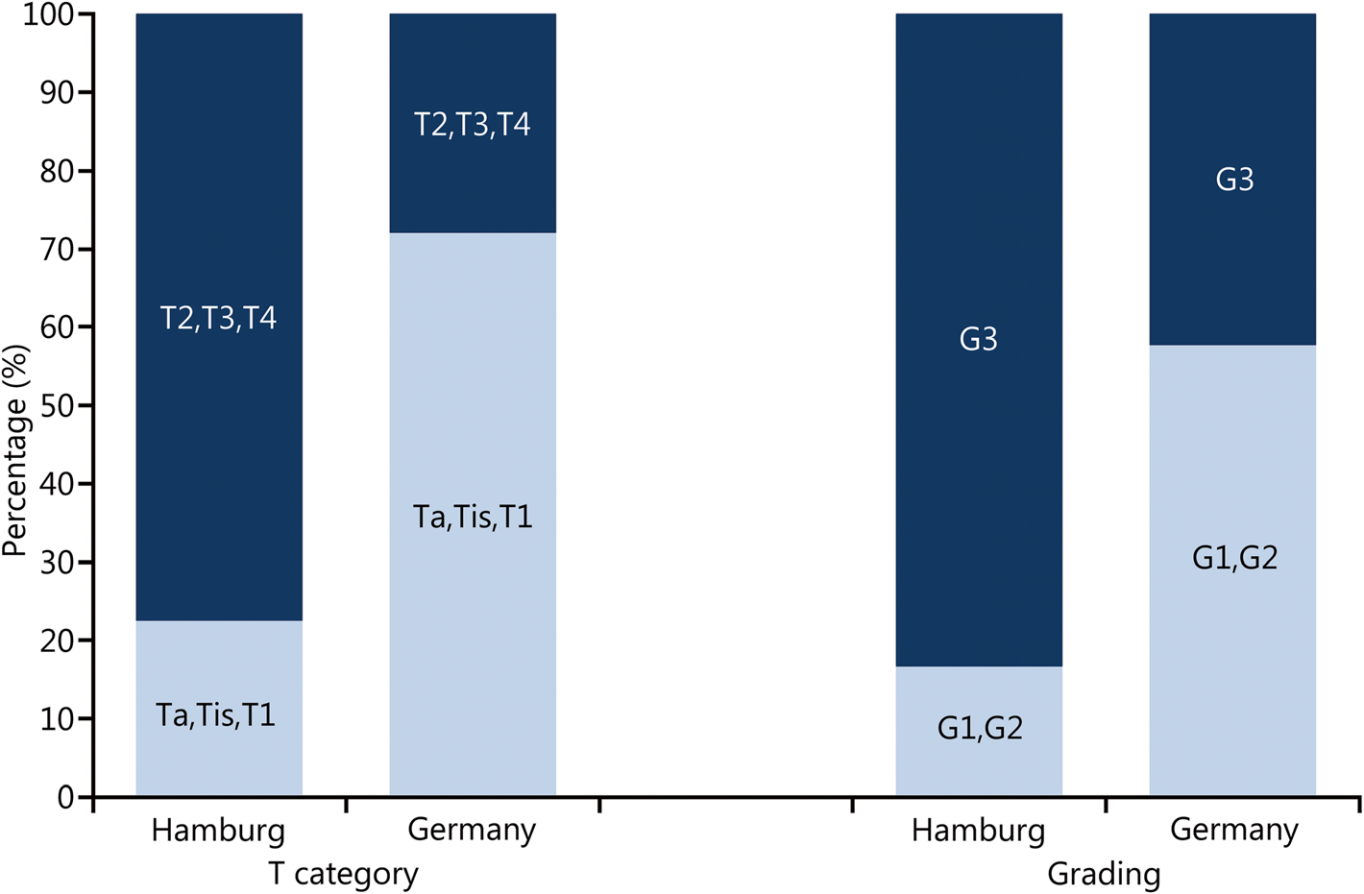
Histopathological findings for T categories and grading. Spinal cord injury patients (Hamburg data 1998–2019) vs. the general population in Germany (Robert Koch Institute data 1999–2016). T category. *P* < 0.0001; Grading. *P* < 0.0002

The observed tumour entities, T categories and gradings of the 40 SCI patients with bladder cancer are shown in Table 1 and were compared with results from bladder cancer patients in the general population in Germany (RKI data 1999–2016) (Fig. 4).

**Table 1:** T categories and grading of bladder cancer in spinal cord injury patients

T category	Grading (*n*)			
	Gx	G1	G2	G3
≥ pT2				
Transitional cell carcinoma	1	0	1	24
Squamous cell carcinoma	2	0	0	2
Undifferentiated carcinoma	1	0	0	0
pT1				
Transitional cell carcinoma	0	0	1	3
pTa				
Transitional cell carcinoma	0	1	1	2
Squamous cell carcinoma	0	1	0	0

Tis, Ta and T1. Superficial tumours; ≥ T2. Muscle invasive tumours

**Fig. 5:**
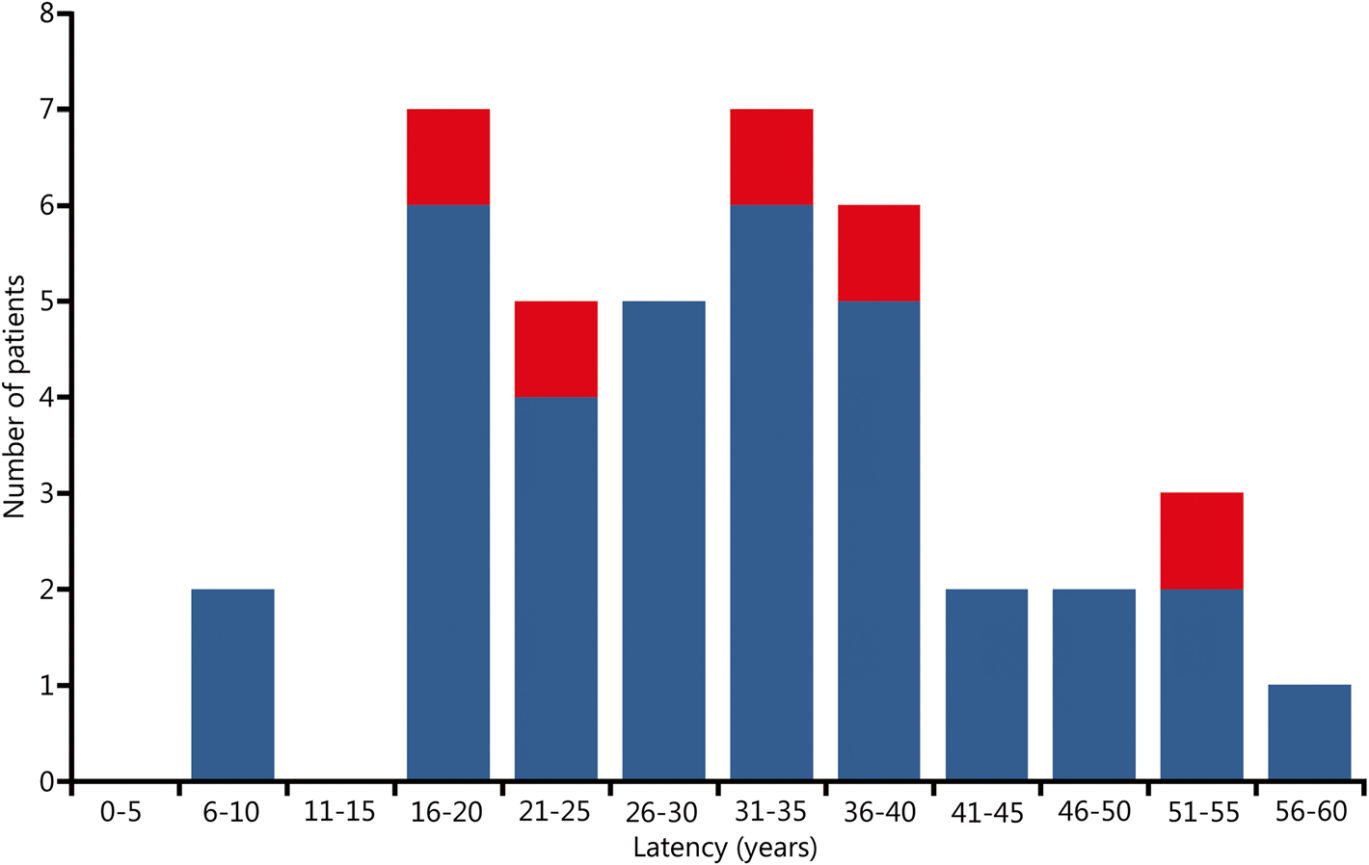
Latency between onset of spinal cord injury and initial bladder cancer diagnosis. Blue. Transitional cell carcinoma; Red. Squamous cell carcinoma

The median time between the onset of SCI and the time of initial tumour diagnosis (latency period) was 32.0 years. Specifically, for patients with transitional cell carcinoma the time was 32.0 years and for patients with squamous cell carcinoma the time was 33.0 years (non-significant difference, *P =* 0.7988; Fig. 5). Patients with an SCI above the sacral micturition centre (UMNL) with detrusor overactivity had a median latency of 29.0 years, while patients with SCI below the sacral micturition centre (LMNL) with acontractile detrusor function had a median latency of 38.0 years (*P =* 0.0069).

The vast majority of the patients with bladder cancer used triggered reflex micturition or intermittent catheterisation for bladder management. The total latency time, i.e.*,* the sum of the latencies of every patient from SCI to initial diagnosis of bladder cancer of all 40 patients was 1251 years. The duration (period of time) in which 8 patients used indwelling transurethral or suprapubic catheters for bladder management was 15.5 years. This corresponds to only 4.45% of the sum of the total latency periods.

The prognosis of the 40 SCI patients with bladder cancer was very poor. The median survival for all patients was 12.0 months (range: 1–177 months). Patients (*n =* 34) with transitional cell carcinoma showed a median survival of 15.0 months with a mean of 37.4 months (range: 1–177 months). The 5 patients with squamous cell carcinoma showed a median survival of only 4.0 months and a significantly (*P =* 0.0141) worse prognosis. One patient showed an undifferentiated carcinoma, with a survival of 1.0 month. After 1 year, 21 of the 40 patients had died, and after 2 years a further 4 had succumbed to the cancer.

In 25 patients a radical cystectomy with curative intention was performed. The median survival was 13.0 months.

## Discussion

### Frequency and mortality

The Center for Cancer Registry Data of the RKI in Berlin, Germany [8] published the standardized disease rate for bladder tumours (International Statistical Classification of Diseases and Related Health Problems, ICD 10: C67) for Germany in 2014 as 18.2/100,000 for males and 4.9/100,000 for females. The “Globocan Project” of the World Health Organisation estimated the age-standardized incidence of bladder cancer (ICD 10: C67) for the world in 2018 at 5.7/100,000 for both sexes, 9.6/100,000 for males and 2.4/100,000 for females [9].

Unfortunately, there are no reliable data on the frequency of SCI in Germany or, to the best of our knowledge, in any other country, so that the basis for calculating an “SCI-specific” disease rate is unavailable. The “incidences” given in most studies are only frequency data, which refer to different observation periods (2 to 47 years) and extremely different populations “at risk” (62 to 43,561 SCI patients). A direct comparison with epidemiological data of the general population is not allowed from a statistical point of view. Consequently, the question of the frequency of bladder cancer in patients with accidental or disease-related SCI has been repeatedly and controversially discussed [5].

Even the data from the only population-based studies, both conducted in Taiwan Province, China, do not allow for a valid determination of bladder cancer incidence in long-term paraplegic patients due to a maximum observation period of 13 years [10] and a maximum follow-up of 11 years [11]. In the Hamburg patient collective, only 2 of 40 patients developed bladder cancer after less than 15 years of paralysis.

However, it can be assumed as certain that the risk of SCI patients dying of bladder cancer is significantly increased. According to a large U.S. mortality study of 99 SCI patients who died of bladder cancer [12], the standardized mortality ratio (SMR) for SCI patients of any sex was 6.69 (95% confidence interval (CI) 5.44–8.14), for male patients was 5.96 (95% CI 4.71–7.44) and for female patients was 12.21 (95% CI 7.56–18.66). The mortality of bladder cancer was, thus, 6.7-times higher than that in the general US population.

In a study from England and Wales [13] on 207 females with SCI, the odds ratio (*OR*) of dying of bladder cancer was 12.0 (95% CI 1.46–99.70). Groah et al. [14] calculated an age- and gender-standardized SMR for paraplegic patients compared with the normal population of 70.6 (95% CI 36.9–123.3), while Stonehill et al. [15] reported a disease-specific mortality rate of 2.9%.

The frequency of bladder cancer in SCI patients, which is controversial on the one hand, and the increased mortality rate, which is considered certain on the other hand, indicate the difficulties that have to be overcome in assessing the causal relationship.

The most important indicators/arguments for and against a causal link will be presented and discussed below.

### Age – early onset of the disease

While the RKI [8] published a median age of onset for bladder cancer of 77 years for females and 74 years for males for the general population in Germany in 2013, the median age reported for SCI patients in a review by Welk et al. [16] ranged from 48 to 61 years (mean value 54.2 years, *n =* 302). In a recent meta-analysis by Ismail [4] (range 48–57 years, mean value 56.1 years, *n =* 332) and Gui-Zhong and Li-Bo [3] (mean value 50 years, 95% CI 45–55 years, *n =* 301) the data were, as expected, in the same range.

In accordance with these data, which are based on approximately 300 SCI patients, the mean age at the time of initial tumour diagnosis was 54.5 years (men 55.0 years and women 51.0 years) in the 40 SCI patients in the Hamburg study (Fig. 1).

Due to the different data sets (“raw/crude” frequency data in the studies vs. incidence) a direct statistical comparison of the age of the tumour patients in the study population compared with the general population in Germany is not possible. However, a comparison of the frequency of the initial diagnosis of bladder cancer in the different age groups among all (male) SCI patients treated in the Hamburg trauma hospital during the observation period with the incidence rate of the (male) general population in Germany (RKI data, Fig. 2) clearly demonstrates differences.

In the case of occupation-related cancers, it is already commonly accepted to evaluate a clear shift in the onset of the disease (“left shift”) to a younger age, compared with the general population, as a convincing indication of a causal relationship in the assessment of occupational cancer in Germany [17]. Even if the underlying pathomechanism that leads to bladder cancer in the presence of a neurogenic bladder dysfunction is not yet known, a relevant younger age at the onset of the disease compared with the general population should be regarded as an important indicator for the recognition of the causal relationship in SCI patients suffering from bladder tumours (Table 2).

**Table 2:** Matrix for assessing the risk factors for the causal relationships between spinal cord injury and bladder cancer

Indicators	Value of the arguments					
	Recognition impossible	Strong counter argument	Weak counter argument	Neutral	Weak pro-argument	Strong pro-argument
Tumour characteristics						
Early onset of bladder cancer (compared with onset in the general population)	–	–	0 years	1–10 years	11–20 years	> 20 years
Latency period (paralysis duration)	< 5 years	–	5–10 years	–	≥11 years	–
Tumour extent (T category) at initial diagnosis	–	–	–	Tis, Ta, T1	≥ T2	–
Tumour type	–	–	–	Transitional cell carcinoma, low grade	Transitional cell carcinoma, high grade or other tumour types	Squamous cell carcinoma
Medical treatment						
Permanent catheter (indwelling catheter, suprapubic catheter)	–	–	–	No catheter	< 5 years	> 5 years
Radiotherapy cervix cancer	–	> 30 years ago	10–30 years ago	< 10 years ago	–	–
Radiotherapy prostate cancer, rectal cancer	–	–	–	yes	–	–
Cyclophosphamide treatment (total dose)	–	≥ 50 g	20–49 g	< 20 g	–	–
Smoking habits						
Never-smoker	–	–	–	–	Yes	–
Smoker till onset of bladder cancer	–	≥ 30 py	10–29 py	< 10 py	–	–
Ex-smoker for 1–9 years	–	≥ 45 py	14–44 py	< 14 py	–	–
Ex-smoker for 10–19 years	–	≥ 45 py	24–44 py	< 24 py	–	–
Ex-smoker for 20–24 years	–	≥ 70 py	25–69 py	< 25 py	–	–
Ex-smoker for ≥25 years	–	–	–	Yes	–	–
Spinal cord injury related factors						
Type of bladder paralysis	–	–	–	UMNL, LMNL	–	–
Urinary tract infections	–	–	–	Yes, regardless of frequency	–	–
Bladder stones	–	–	–	Yes	–	–

Tis, Ta and T1. Superficial tumours; ≥ T2. Muscle invasive tumours

*py* Pack years, *UMNL* Upper motor neuron lesion, paralysis above the medullary conus, *LMNL* Lower motor neuron lesion, acontractile or flaccid paralysis of the bladder

### Duration of SCI

In the Hamburg study population, the average time (Fig. 3) between the onset of SCI and initial tumour diagnosis was 32.0 years. A current meta-analysis of the available case series [3] reported a mean duration of SCI of 24 years (95% CI 21–27 years, heterogeneity factor *I*^2^ = 92.7%), while another meta-analysis [4] reported a mean duration of SCI of 24.9 years.

Vereczkey et al. [18] determined the duration of paralysis as the only risk factor among the risk factors they investigated (duration of SCI, smoking, permanent catheter use, urinary tract infections or bladder stones) for the development of bladder cancer in SCI patients using both univariate and multivariate analyses (*P =* 0.0006).

The mortality study by Nahm et al. [12] determined (with a mean interval between the onset of SCI and bladder cancer of 23.9 years (standard deviation 8.5 years)) an SMR for bladder cancer standardized by age and sex after 1–9 years of paralysis of 1.42 (95% CI 0.57–2.93), after 10–19 years of paralysis of 3.96 (95% CI 2.34–6.25) and after more than 20 years of paralysis of 17.83 (95% CI 14.00–22.39). This significant increase in the risk of dying from bladder cancer with increasing duration of SCI indicates a direct relationship between these two factors (Table 2).

### T category

The statistical comparison of the frequency of superficial tumours (Tis, Ta and T1) and muscle invasive tumours (≥ T2) in the Hamburg study population with the data of the RKI showed a highly significant increase (*P* < 0.0001) in the incidence of muscle invasive tumours in SCI patients (Fig. 4).

The proportion of muscle invasive tumours at initial diagnosis was between 50 and 100% in all published case series. A systematic review [4] calculated the frequency of muscle invasive tumours in paraplegic patients at 76.7%; in the Hamburg patient population it was 78.1% (for comparison, the normal German population is 29.8%).

The considerably more frequent primary occurrence of locally advanced, muscle invasive tumours (≥ T2) in comparison with the general population also indicates a causal association between SCI and bladder cancer (Table 2).

### Grading

With regard to tumour grading, almost no studies are available for paraplegic patients. In the Hamburg study population, however, significant differences in the cancer grading, compared with the general population in Germany (RKI 1999–2016) were found (Fig. 4). Poorly differentiated tumours (G3) occurred more frequently (*P* < 0.0002) in patients with paraplegia compared with better differentiated tumours (G1 and G2) (Table 2).

### Tumour entity

In Western industrial nations, the frequency of squamous cell carcinoma of the urinary bladder is only 3–6% [19]. A meta-analysis [3] of the available studies indicates the frequency of squamous cell carcinoma in patients with paraplegia to be 36.8% (95% CI 31.6–42.5; *I*^2^ = 32.3%). Two studies from German-speaking regions showed the frequency of squamous cell carcinoma in SCI patients to be 17% [20] and 15.6% (Hamburg study population) [6].

Taken together, the incidence of poorly differentiated tumours and/or squamous cell carcinomas in SCI patients supports the assumption of a causal relationship between the two (Table 2).

### Bladder management

Chronic permanent bladder drainage via suprapubic or transurethral catheters is considered a proven risk factor for the development of bladder cancer in non-SCI patients.

Groah et al. [14] calculated a relative risk (*RR*) for SCI patients with permanent indwelling catheters of 4.9 (95% CI 1.3–13.8, *P =* 0.02), compared with permanent catheter-free bladder management. A catheter use of more than 20 years increased the *RR* compared with a catheter use of up to 10 years by a factor of 4.6 (95% CI 1.5–14.0). Stonehill et al. [15] stated a *RR* for SCI patients with permanent catheters of 12.8. The risk analysis of Vereczkey et al. [18] is of considerable importance for the weighting of the risk factor “urinary diversion through the permanent catheter”. It showed a significant (*P =* 0.0006) predictive value for SCI patients with an indwelling catheter drainage of the urinary bladder over a period of more than 10 years in univariate analysis, but a significant influence was no longer detectable in multivariate analysis.

In our own study, the times of urinary diversion via permanent catheters played only a very minor role (4.45% of the sum of the added-up latency times). Therefore, indwelling catheterization cannot be considered as a decisive risk factor for bladder cancer in SCI patients. Furthermore, this fact reflects the high level of the (neuro-urological) care of SCI patients in Germany: Only a small number of these patients are treated with permanent indwelling catheter drainage of the urinary bladder. However, this in no way disproves the importance of permanent catheter drainage of the urinary bladder for the development of bladder cancer. As a consequence of the above findings, the European Association of Urology guidelines, “Urological Infections 2015” [21] recommend annual tumour screening after 10 years of use of an indwelling bladder catheter (grade of recommendation C). Paralyzed Veterans of America also recommend more frequent cystoscopic controls for SCI patients with permanent catheters [2].

### Type of bladder paralysis

In the Hamburg study, 35 patients with spinal reflex bladder (detrusor overactivity) with UMNL (paralysis above the medullary cone) and 5 patients with acontractile detrusor function with LMNL (areflexia or flaccid paralysis of the urinary bladder) were evaluated.

The data were not able to determine if different neurogenic types of impaired bladder function could be used as differentiating risk parameters for the development of cancer.

### Urinary tract infections

SCI patients with neurogenic bladder dysfunction are more likely to suffer from urinary tract infections (UTI) than people with undisturbed bladder function [22]. In some studies, both SCI and non-SCI patients with recurrent UTI had a twofold increased risk of developing bladder cancer [23]. To the best of our knowledge, prospective studies based on microbiologically-verified data on the relationship between UTI and the occurrence of urinary bladder tumours do not exist.

In a retrospective data collection in SCI centres in German-speaking regions [20], 24.3% of patients with bladder cancer reported more than 10 UTI per year and another 40.6% suffered from chronic UTI. In the Hamburg study population, 14 of 32 patients (43.8%) reported 3 or more UTI episodes per year (unpublished data). However, the cited studies are based only on anamnestic data and not on microbiologically confirmed findings.

In principle, the data available in the scientific literature on the association of UTI, SCI and bladder cancer is insufficient. The main problem is the distinction between symptomatic UTI and “asymptomatic bacteriuria”, which frequently occurs in paraplegics [24]. The frequency of UTI is therefore not a reliable criterion for the evaluation of the causal relationship.

However, a recently published country-wide Danish study connecting the data of 333 patients with squamous cell carcinoma with their prescription of antibiotics targeting UTI, describes a clear dose-response relationship between UTI and squamous cell carcinoma of the urinary bladder. The highest risk group, i.e., 20 and more prescriptions of UTI specific antibiotics, had an *OR* of 14.4 (95% CI 7.9–26.4) for squamous cell carcinoma. However, UTI-specific antibiotic prescription was not associated with transitional cell carcinoma (*n =* 11,029; *OR* 1.13; 95% CI 0.97–1.32) [25].

### Urinary bladder stones

The hazard ratio (*HR*) with regard to the risk of bladder stones was 10.5 (*P* < 0.0005, 95% CI 4.0–27.5) in SCI patients with a suprapubic catheter and 12.8 (*P* < 0.0005, 95% CI 5.1–31.9) in patients with a permanent transurethral catheter [26]. In a study of non-SCI patients, it was found that bladder stones, in contrast to kidney stones, increased the risk of bladder cancer (*RR* 1.8, 95% CI 1.1–2.8) [23].

In SCI patients with bladder cancer, significantly higher bladder stone rates have been reported in some cases [15]. Groah et al. [14], on the other hand, did not find a correlation between bladder cancer and bladder stones using a multivariance analysis.

Due to the currently inconsistent data, it is not reasonable to consider bladder stones as an independent risk factor.

### Bladder cancer in spina bifida (congenital paraplegia)

Spina bifida is predominantly associated with congenital paraplegia. So far, only one systematic review has been published on this very specific patient population, based on 52 patients from 28 case reports and case series [27]. Spina bifida patients also develop advanced bladder tumours at a much earlier age than the general population. Indeed, the mean age at initial diagnosis of bladder cancer was 41 years (range 13–73 years). Overall, 71% of spina bifida patients had a stage III or IV bladder cancer at initial diagnosis. The overall survival rate was 48.5% after 1 year and 31.5% after 2 years. The youngest age of 13 years at initial diagnosis of bladder cancer in this group confirms minimum paralysis duration of more than 10 years. These characteristics of patients with congenital paraplegia impressively confirm the data of acquired SCI.

### Other trauma-independent risk factors for bladder cancer

Other trauma-independent risk factors relevant for the evaluation of a causal relationship between SCI and bladder cancer are smoking [5, 6, 28–32], radiotherapy [33–37] and chemotherapy with cyclophosphamide [38–40]. Additional file 1 presents more details on this issue.

### Aids for the evaluation of a causal relationship between long-term SCI and bladder cancer

The previously mentioned risk factors have been taken into account in Table 2, which was developed to aid decision makers in answering the question of whether the cause of the bladder cancer disease is due to the SCI or not. This is an important issue in cases where the granting of financial rewards or health services is depending on the cause of the disease.

### Future developments

The work of Manach et al. [41] describes for the first time, albeit in a small number of cases, molecular biological differences in 20 bladder cancers of neuro-urological patients compared with 40 bladder cancers of other patients. Their results indicated that GATA3 expression showed a high association with the luminal subtype only in bladder cancers without neuro-urological history. This distinction of molecular subtypes with different expression of protein markers (luminal and basal type) could have therapeutic consequences in the future.

## Conclusions

Bladder cancer in SCI patients occurs at a significantly younger age compared with the able-bodied population, and has a considerably worse prognosis. The increased risk of bladder cancer begins after 10 years of paralysis. The presented algorithm is an important aid in everyday clinical practice for assessing the correlation between SCI and bladder cancer. The algorithm should also help military and civil experts to weigh the most important factors that point to or against a causal relationship between the two. Indeed, a recently published review by Liu and Welk [42] showed a lack of consensus on how to appropriately screen patients with neurogenic bladder for bladder cancer. We support the view of Liu and Welk that “good history and physical exam of patients with neurogenic lower urinary tract disease is appropriate in this population to look for potential signs of symptoms of bladder cancer.”

## Supplementary Information

**Additional file 1.** Details of other trauma-independent risk factors for bladder cancer.

## Data Availability

The datasets used and/or analysed during the current study are available from the corresponding author on reasonable request.

## Abbreviations

CI: Confidence interval
IARC: International Agency for Research on Cancer
ICD: International Classification of Diseases and Related Health Problems
LMNL: Lower motor neuron lesion
RKI: Robert Koch Institute
RR: Relative risk
SCI: Spinal cord injury
SMR: Standardized mortality ratio
UMNL: Upper motor neuron lesion
UTI: Urinary tract infections
