# Supplementary material for: Urinary bladder cancer as a late sequela of traumatic spinal cord injury
Source: Mil Med Res. 2021 Apr 29;8:29. doi: 10.1186/s40779-021-00322-7 (PMC8082770; doi:10.1186/s40779-021-00322-7)
Supplement: Supplementary file 1 — Additional file 1. Details of other trauma-independent risk factors for bladder cancer. [file 40779_2021_322_MOESM1_ESM.docx]

# Additional files

**Additional file 1.** Details of other trauma-independent risk factors for bladder cancer

**Smoking**

Studies suggest that approximately 50% of men who develop bladder cancer develop this tumour as a result of smoking habits [28]. A recent, large cohort study indicated that this also applies to women [29]. A particular risk feature of smoking and the development bladder cancer is that in all the major studies smoking does not exceed the magnitude of a relative risk (*RR*) of 6 [30].

The influence of smoking on the risk of bladder cancer is difficult to assess. This is shown, among others, by the risks, which may differ considerably from one study to the next. For example, the doubling risk (*RR* = 2) of a smoker of developing bladder cancer is reported in a range of 15 to 38 pack years [6].

The publication of Brennan et al. [30] has turned out to be the most important work on the evaluation of a smoker’s risk of developing bladder cancer given the wide range of results of the major smoking studies. It was used by International Agency for Research on Cancer (IARC [31]) as the basic study for assessing the risk of bladder cancer in men due to smoking. Interestingly, it was shown that the bladder carcinogenic effect of a given number of pack years is higher when the cigarettes were consumed over a long period [32].

**Special features in the assessment of the smoking risk**

Quitting smoking leads directly to a reduction in the risk of bladder cancer. In particular, the authors of the International Agency for Research on Cancer (IARC [31]) found two main points in regard to the time span/interval between quitting smoking and initial diagnosis of bladder cancer with regard to smoking-related risk: In the first 4 years, the risk of bladder cancer decreases by about 30%, while after 20 years, the risk approaches that of a never-smoker but does not reach that level.

This results in the decreasing risk of ex-smokers shown in the matrix (Table 2). Studies that explicitly investigate a possible connection between smoking and bladder cancer in SCI patients are rare. Vereczkey et al. [18] could not prove a significant effect in univariate and multivariate analyses.

Groah et al. [14] found that the *RR* for an SCI patient (with permanent catheter drainage) to develop bladder cancer remained almost unchanged when adjusted to the smoker status (RR 4.9; 95% CI 1.3–13.8 vs. RR 4.4; 95% CI 2.5–7.8). Except for these two studies, all other studies hardly dealt with this topic or not at all.

The conclusions of Vereczkey et al. [18] and Groah et al. [14] are consistent with the general life experience that a paraplegic smoker who develops bladder cancer at an average age of 55 years has a lower smoking burden than a person who develops bladder cancer at an average age of 75 years, and thus, has accumulated a further 20 years of smoking.

On the basis of the available data, it must be concluded that the extent of the influence of smoking on the risk of bladder cancer in patients with spinal paralysis cannot be scientifically justified (Table 2).

**Radiotherapy**

Boice et al. [33] showed in 1985, on the basis of a study with 182,040 women who had received radiotherapy for cervical carcinoma, that the risk of developing bladder cancer increased with the latency period. After more than 30 years, the RR was particularly high at 8.5.

Neugut et al. [34] observed a 1.3-fold increased risk 5 to 8 years after therapy and a 1.48-fold increased risk after more than 8 years in a large group of patients (n = 34,889) who had received radiation therapy for prostate cancer. These data are consistent with the data from the studies of Kaldor et al. [35], Liauw et al. [36], and Moon et al. [37].

In summary, it can be stated that the radiotherapy of cervical carcinoma in earlier decades is a considerable competing risk factor for the development of bladder cancer. Radiotherapy of prostate cancer and intestinal tumours, on the other hand, does not appear to be relevant due to the lower risk increase (Table 2).

**Chemotherapy with cyclophosphamide**

In a review, 21% of 148 cyclophosphamide-induced secondary tumours were described as bladder cancer, which occurred after a mean latency period of 52 months [38].

In a study of 19,040 tumour patients with a survival time of more than 5 years, a 9-fold increase in the incidence of bladder cancer was observed in 170 patients receiving cyclophosphamide therapy [39]. In a study of 6,171 patients with non-Hodgkin’s lymphomas treated with cyclophosphamide, 31 bladder cancer cases were observed. The risk increases from 2.4-fold (total dose < 20 g) to 14.5-fold (> 50 g) [40] (Table 2).
